# Analysis of nuclear and mitochondrial genes in patients with pseudoexfoliation glaucoma

**Published:** 2008-01-10

**Authors:** Khaled K. Abu-Amero, Thomas M. Bosley, Jose Morales

**Affiliations:** 1 Mitochondrial Research Laboratory, Genetics Department, King Faisal Specialist Hospital and Research Centre, Riyadh, Saudi Arabia; 2 Neuroscience Department, King Faisal Specialist Hospital and Research Centre, Riyadh, Saudi Arabia; 3 Glaucoma Division, King Khaled Eye Specialist Hospital, Riyadh, Saudi Arabia; 4 Neuro-ophthalmology Division, King Khaled Eye Specialist Hospital, Riyadh, Saudi Arabia

## Abstract

**Purpose:**

Pseudoexfoliation glaucoma (PEG) is the most prevalent secondary open angle glaucoma occurring worldwide. The search for a genetic cause in PEG has been largely unsuccessful despite evidence of hereditary transmission.

**Methods:**

The nuclear genes *MYOC*, *OPTN*, *WDR36*, *CYP1B1*, *OPA1*, and *OPA3* were sequenced in patients with PEG. The entire mitochondrial DNA (mtDNA) coding region was also sequenced, relative mtDNA content was investigated, and mitochondrial respiration was assessed.

**Results:**

No novel or previously reported mutations were present in the nuclear genes *MYOC*, *OPTN*, *CYP1B1*, *WDR36*, *OPA1*, or *OPA3* in 29 PEG patients. Twenty-six patients (89.7%) had no pathological or potentially pathological mtDNA mutation(s); however, three patients (10.3%) had potentially pathologic mtDNA nucleotide changes not found in controls. PEG patients did not differ significantly from controls in relative mitochondrial content (p=0.98) or in mitochondrial respiratory activity (p=0.18).

**Conclusions:**

These PEG patients had no mutations in nuclear genes associated with other types of glaucoma or other inherited optic neuropathies, and there was little evidence of mitochondrial abnormalities. These results imply that the nuclear genes and mitochondrial parameters evaluated here are less important determinants of PEG than other factors related to the presence of pseudoexfoliation material.

## Introduction

Pseudoexfoliation syndrome (PES) is characterized by deposits of grayish-white material throughout the anterior segment that are generally most easily recognizable around the pupillary border and over the lens surface. PES is frequently associated with pseudoexfolation glaucoma (PEG), which often has a more serious clinical course and worse prognosis than the more common primary open angle glaucoma (POAG). Cataract and central retinal vein occlusion occur more often, and cataract surgery is sometimes complicated by fragile zonules and lens capsule as well as poor pupillary dilation. PES may be a systemic condition [1,2] associated with systemic hypertension, transient ischemic attacks, stroke, and myocardial infarction [3,4], and recent studies have strengthened claims that inheritance plays a potential role [5,6]. In fact, nearly all reported PES pedigrees suggest maternal transmission, raising the possibility of mitochondrial inheritance [7,8]. Matrilineal inheritance, variable expression, late age of onset, multisystem involvement, and reduced amount of mitochondria in iris tissue are all features consistent with mitochondrial involvement [5,7,8].

In a previous study of adult onset POAG, we reported an increased frequency of nonsynonymous (NS) mitochondrial DNA (mtDNA) sequence changes and decreased mitochondrial respiration in patients compared to controls [9]. These results raise the question of whether abnormal mitochondria play a role in other types of glaucoma as well. The aim of the current study was a similar evaluation of PEG patients for the presence of mitochondrial abnormalities and nuclear gene mutations associated with various types of glaucoma (*MYOC*, *OPTN*, *WDR36*, and *CYP1B1*) and certain inherited optic neuropathies (*OPA1* and *OPA3*) [10-12].

## Methods

### Patient enrollment

Patients were eligible for inclusion in this study if they had in at least one eye: (1) characteristic pseudoexfoliation grayish-white particles over the pupillary border and/or surface of the lens noticed before or after pupillary dilation; (2) glaucomatous optic disc changes diagnosed by vertical excavation measuring at least 0.65 cup-to-disc (c/d) ratio with localized thinning of the rim in one or both vertical poles; and (3) glaucomatous visual field loss including nerve fiber bundle defects (nasal step, arcuate scotoma, paracentral scotoma) or advanced visual field loss (central and/or temporal island of vision). Exclusion criteria included a history of other possible optic neuropathies affecting either eye, significant visual loss in both eyes not associated with glaucoma, lack of adequate visualization of the fundus for disc assessment, or refusal to participate.

Patients were selected from the Glaucoma Clinic at King Khaled Eye Specialist Hospital after examination by a glaucoma specialist (J.M.) and after signing informed consent approved by the KKESH-IRB. Records were reviewed, and full ophthalmologic examinations were performed. Patients had either Goldmann manual kinetic perimetry (Haag Streit International, Koeniz-Bern) or Humphrey automated white on white stimulus static perimetry (Humphrey Field Analyzer II, Humphrey Systems, Dublin, California), or both. Optical Coherence Tomography was performed with the OCT3 Unit by Humphrey Systems (San Leandro, CA) on some patients. Fundus photos were obtained using a Zeiss FF 450 system and conventional film. This research followed the tenets of the Declaration of Helsinki. Family members were not evaluated clinically or genetically.

### Control enrollment

Control subjects were blood donors at the King Faisal Specialist Hospital and Research Centre who represented the spectrum of Saudi Arabs and who reported no symptomatic metabolic, genetic, or ocular disorders on an extensive questionnaire about family history, past medical problems, and current health. Health information was obtained from controls only through the questionnaire; none had a physical or ophthalmological examination. The control group for mitochondrial DNA (mtDNA) sequencing consisted of 159 individuals (106 males and 53 females, mean age 46.3±3.8 years); for relative mtDNA content, 28 different individuals (19 males and 9 females, mean age of 59.2±3.3 years); and for mitochondrial respiration testing, 50 different individuals (39 males and 11 females, mean age 59.0±6.7 years). Information of family history was obtained from participating members. All patients and control subjects were of Middle Eastern Arabic origin.

### Sample collection and DNA extraction

Ten ml of peripheral blood were collected in EDTA tubes from all participating individuals after obtaining their written consent. DNA was extracted from whole blood samples of all PEG patients and controls using the PUREGENE DNA isolation kit from Gentra Systems (Minneapolis, MN).

### Isolation of lymphocytes from peripheral blood and preparation of cell suspension

Blood (5 ml) was diluted with phosphate buffered saline (PBS) at a ratio of 1:1 within 1 h of extraction and slowly layered onto a 15 ml screw cap tube containing 4.5 ml Ficoll-Hypaque separating solution. The tubes were centrifuged for 20 min at 1,000 xg after which the lymphocyte-containing layer was collected into a new centrifuge tube using a sterile pipette. The lymphocytes mix was then diluted in 10 ml PBS and centrifuged for 10 min at 660 xg. The supernatant was discarded, 5 ml of hypotonic PBS lysing buffer was added, the pellet was mixed gently in this buffer, and the mixture was allowed to sit for about 45 s. Five ml of 2X NaCl solution was added. The mixture was gently pipetted and then centrifuged at 600 xg for 10 min. The supernatant was discarded, and the pellet was suspended in RPMI-1640 medium (Gibco, Invitrogen Corporation, Carlsbad, CA) supplemented with L-glutamine. The optical density (OD 660) of the lymphocyte suspension was adjusted to 0.20, which is equivalent to a cell density of approximately 5x10^5^ cells/ml. Using this protocol, cell viability assessed by 0.2% trypan blue was 96±2%. These cells were used for mitochondrial respiration testing.

### Sequence analysis of nuclear genes

The coding exons, exon-intron boundaries, and promoter regions in the *MYOC*, *OPTN*, *CYP1B1*, and *WDR36* genes were amplified by PCR from genomic DNA for all patients and controls and subjected to direct sequencing as described previously [13]. The 31 coding exons, exon-intron boundaries, and promoter regions of the *OPA1* gene were amplified by PCR from genomic DNA for all patients and subjected to direct sequencing as described previously [14]. Similarly, the entire *OPA3* gene was sequenced in all patients using the protocol described previously [12].

### DNA amplification and sequencing

The entire coding region of the mitochondrial genome was amplified in all patients and controls in 24 separate polymerase chain reactions (PCRs) using single set cycling conditions as detailed elsewhere [15]. Primers were used to amplify the entire coding region of the mitochondrial genome except the D-loop [16]. PCRs were run under the following PCR conditions: 20 ng of each DNA sample in a 50 ml PCR reaction mixture containing 200 mM dNTP, 0.2 mM of each primer-pair, 1 unit of TaqDNA polymerase, 50 mM KCL, 1.5 mM MgCl_2_ and 10 mM Tris-HCl (pH 8.3). Polymerase chain reaction was performed for 35 cycles and 55 °C annealing temperature in a GeneAmp 9700 PCR (Perkin-Elmer, Foster City, CA). PCR-Primers were designed to avoid amplifying mtDNA-like sequences in the nuclear genome. Each successfully amplified fragment was directly sequenced using the same primers used for amplifications and the BigDye Terminator V3.1 Cycle Sequencing kit (Applied Biosystems, Foster City, CA). Samples were run on the ABI prism 3100 sequencer (Applied Biosystems). A similar sequencing protocol was followed for the nuclear genes.

### Sequence analysis of the mitochondrial DNA coding region

The full mtDNA genome was sequenced except for the D-loop. Sequencing results were compared to the corrected Cambridge reference sequence [16]. All fragments were sequenced in both forward and reverse directions at least twice for confirmation of any detected variant. All sequence variants from both PEG patients and controls were compared to the Human Mitochondrial Genome Database (Mitomap) [17], GenBank, and Medline listed publications. Reported synonymous or NS polymorphisms used mainly for haplogroup analysis were excluded from further consideration [18].

### Prediction of pathogenicity

Pathologic characteristics of each remaining nucleotide change in both PEG patients and controls were assessed according to a combination of: standard criteria [19]; an evaluation of interspecies conservation using the PolyPhen database, and the Mamit-tRNA website when a sequence variant is detected in the tRNA region; assessment of the possible impact of an amino acid substitution on three-dimensional protein structure using the Protean program, part of the LASERGENE V.6 software (DNASTAR, Inc. Madison, WI), which predicts and displays secondary structural characteristics; and assessment of the possible effect of the mtDNA change on protein function using PolyPhen [20]. Therefore, a NS sequence change was considered pathologic if it met al.l of the following criteria, when applicable: (1) it was not a haplogroup-determining polymorphism; (2) it was not reported in mitochondrial databases or available literature as an established polymorphism; (3) it was not found in at least 100 controls of matching ethnicity; (4) it changed a moderately or highly conserved amino acid; (5) Protean predicted an alteration of protein structure; and (6) it was assessed as possibly or probably pathologic by PolyPhen. For previously reported NS nucleotide changes, consideration was given to pathologic status determined by others and by mitochondrial databases in addition to these criteria.

### Determination of relative mitochondrial DNA content

A competitive multiplex PCR was performed with two simultaneous primer sets as described previously [21]. This technique has been applied successfully to a variety of tissues [22,23], including blood of patients with LHON [24] and other spontaneous optic neuropathies [9,25,26]. One primer-pair was designed to amplify a 450 bp fragment of the ND1 mitochondrial gene (forward primer sequence 5′-ACA TAC CCA TGG CCA ACC TC-3′ and reverse primer sequence 5′-AAT GAT GGC TAG GGT GAC TT-3′), while a second primer-pair was used in the amplification of a 315 bp fragment of the β-actin nuclear gene (forward primer sequence 5′-ATG TTT GAG ACC TTC AAC AC-3′ and reverse primer sequence 5′-CAT CTC TTG CAC GAA GTC GA-3′), which served as an internal control. Control and patient PCRs were run simultaneously under the following PCR conditions: 20 ng of each DNA sample in a 50 ml PCR reaction mixture containing 200 mM dNTP, 0.2 mM of each of the ND1 primer-pair, 0.6 mM of each of the β-actin primer-pair, 1 unit of Taq DNA polymerase, 50 mM KCL, 1.5 mM MgCl_2_ and 10 mM Tris-HCl (pH 8.3). Polymerase chain reaction was performed for 25 cycles in a GeneAmp 9700 PCR system (Perkin-Elmer). One ml of SYBR® Green I stain was added to the reaction mixture in the last cycle to label the PCR products. PCR products were separated on 1% agarose gel at 100 V for 1 h, and intensity of the two bands was quantified by the use of gel imager (Typhoon 9410; GE Amersham Biosciences, Schenectady, NY). The ratio of ND1 to β-actin was determined for each patient and control by dividing the fluorescence intensity of the ND1 band by the intensity of the β-actin band.

### Measurement of mitochondrial respiration

Resazurin is a redox-active blue dye that fluoresces and turns pink when reduced. It competes with oxygen for electrons in a standard preparation of circulating lymphocytes, and the resulting change in fluorescence (corrected for background and protein concentration) reflects respiration. Lymphocytes from patients and controls were incubated with 6 μM resazurin without and with mitochondrial inhibition by amiodarone 200 μM, and the fluorescence intensity resulting from resazurin reduction was monitored spectrofluorimetrically over time. Mitochondrial respiratory activity (MRA) was calculated as the difference between uninhibited and inhibited measurements at 240 min. Measurements taken in triplicate were averaged and normalized for protein concentration and background activity as described previously [27]. Mitochondrial metabolic activity has been assessed using Resazurin in synaptosomes from spinal cord-injured animals [28], neonatal rat cerebellum [29], and in isolated yeast mitochondria [30]. The current technique has been validated in systemic mitochondrial disorders [27] including LHON-like optic neuropathies [25].

### Statistical methods

All statistical analyses were performed using SPSS for Windows version 15.0 (SPSS Inc., Chicago, IL). Snellen visual acuities were converted to ordinal values. Statistical comparisons included bivariate correlation, independent samples *t*-test, and Fisher's Exact Analysis.

## Results

### Clinical information

Table 1 details the clinical characteristics of 29 unrelated PEG patients (mean age 68.58 ±9.95 SD years; 24 males and five females) who met inclusion and exclusion criteria. Fifteen patients had bilateral and 14 had unilateral pseudoexfoliation material. Seventeen patients had open anterior chamber angles and 12 patients had undergone laser iridotomies to treat a narrow angle component. Fifteen patients had a visual acuity of 20/200 or less in at least one eye. Results of Optical Coherence Tomography testing and fundus photos were consistent with the clinical assessment of optic disc damage (data not shown).

**Table 1:** Clinical characteristics of PEG patients.

**Patient**	**Age**	**Sex**	**PXF side**	**IOP OD**	**IOP OS**	**c/d OD**	**c/d OS**	**VA OD**	**VA OS**	**VF OD**	**VF OS**
1	77	M	Both	24	25	0.75/.7	0.85/.8	20/30	20/20	Inferior arcuate scotoma with nasal step	Inferior arcuate scotoma with nasal step
2	65	M	Both	42	46	0.8/.8	0.9/.9	20/30	20/25	Central island remnant	Central island remnant
3	69	M	Both	35	44	0.75/.7	0.95/.9	20/20	20/400	Central island remnant	Inferior arcuate scotoma with nasal step and split fixation
4	73	M	Right	26	25	0.95/.9	0.8/.8	1/200	20/40	Superior arcuate scotoma + nasal step	Central scotoma
5	71	F	Both	27	22	0.9/.85	0.5/.5	20/100	20/50	Inferior arcuate scotoma with nasal step	Inferior nasal step
6	100	M	Both	19	19	0.9/.8	0.95/.9	20/60	HM	Generalized non-specific depression	Unable
7	79	F	Both	36	40	0.95/.9	0.9/.8	20/50	20/40	Central island remnant	Central island remnant
8	63	F	Left	19	34	0.4/.4	0.95/.9	20/30	4/200	Normal	Central island remnant
9	69	M	Both	20	27	0.4/.4	0.7/.6	20/20	20/30	Normal	Superior and inferior arcuate scotomas with nasal step
10	67	M	Both	30	28	0.9/.8	0.6/.5	20/20	20/25	Central island remnant	Small nasal step
11	71	F	Left	20	30	0.95/.9	0.6/.5	20/100	20/40	Central island	Generalized depression
12	69	M	Both	20	17	0.95/.9	0.8/.8	20/100	20/30	Central island remnant	Non specific diffuse depression
13	62	F	Right	40	20	0.95/.9	0.3/.2	HM	20/40	Temporal island remnant	Normal
14	65	M	Both	44	38	Pros.	0.95/.9	NLP	HM	Unable	Central and temporal island
15	49	M	Right	34	30	0.75/.7	0.95/.9	20/30	20/125	Inferior arcuate scotoma with nasal step	Dense superior and inferior arcuate scotomas approaching fixation
16	65	M	Both	28	30	0.8/.5	0.5/.5	20/25	20/20	Inferior arcuate scotoma and inferior nasal step	Inferior nasal step
17	60	M	Left	21	46	0.75/.7	0.95/.9	20/25	NLP	Inferior arcuate scotoma and inferior nasal step	Unable
18	72	M	Left	39	40	0.85/.8	0.99/.9	20/50	NLP	Inferior arcuate scotoma with nasal step	Unable
19	60	M	Both	35	17	0.65/.6	0.3/.2	20/60	20/30	Superior and inferior arcuate scotomas	Normal
20	69	M	Both	20	43	0.5/.5	0.95/.9	20/40	HM	Small inferior nasal step	Unable
21	65	M	Right	42	24	0.9/.8	0.85/.8	20/100	20/50	Central island remnant	Superior hemifield defect with inferior paracentral scotoma
22	84	M	Right	21	60	0.9/.8	unable	20/300	NLP	Superior arcuate scotoma with nasal step	Unable
23	57	M	Right	31	22	0.9/.8	0.4/.4	20/25	20/20	Central island remnant	Normal
24	68	M	Right	35	53	0.6/.5	0.95/.9	20/100	HM	Generalized depression	Unable
25	76	M	Left	43	54	0.6/.5	0.95/.9	20/20	20/200	Early inferior nasal step	Central island remnant
26	51	M	Left	18	40	0.4/.4	0.85/.8	20/25	20/20	Normal	Central island remnant
27	67	M	Both	50	22	0.95/.9	0.7/.6	LP	20/30	Unable	Central island remnant
28	81	M	Left	17	33	0.85/.8	0.95/.9	20/60	HM	Central island remnant	Unable
29	65	M	Both	22	50	0.95/.9	0.99/.9	20/80	NLP	Central island remnant	Unable

Age=age in years; IOP=maximum documented intraocular pressure; OD=right eye; OS=left eye; c/d=cup to disk ratio in vertical/horizontal dimensions; VA=visual acuity by Snellen plates; VF=results of Goldmann and/or Humphrey visual field; M=male; F=female; HM=hand motion; LP=light perception; NLP=no light perception

### Sequence analysis of *MYOC*, *OPTN*, *WDR36*, *CYP1B1*, *OPA1*, and *OPA3*

No novel or previously reported sequence mutation was found in *MYOC*, *OPTN*, *WDR36*, *CYP1B1*, *OPA1,* or *OPA3* genes in PEG patients or controls. Patients had no previously reported or novel polymorphisms in any of these genes, and controls had only polymorphisms reported previously [9].

### Sequence analysis of the mitochondrial coding region

The prevalence of NS mtDNA nucleotide changes in PEG patients was not different from controls (Fisher's Exact Analysis p=0.86). Table 2 details the 13 NS mtDNA changes found in PEG patients after excluding all synonymous mtDNA changes, established NS polymorphisms, and NS mtDNA sequence changes relevant primarily to haplogroup designation. Four of these mtDNA sequence changes were novel (not previously reported), and the remaining nine were also found in ethnicity-matched controls. All previously reported nucleotide changes were transitions, while two of the novel changes were transversions and two transitions. None were heteroplasmic. Three novel NS mtDNA sequence changes were considered pathologic using the criteria described in the Methods.

**Table 2:** Nonsynonymous mtDNA sequence changes detected in PEG patients.

**Nucleotide Substitution**	**AA Change**	**Location**	**Base substitution type**	**Controls (%)**	**Novel**	**Interspecies Conservation**	**Protean**	**PolyPhen**	**Summary**
3833 T>A	L176Q	TM domain of ND1 gene	Transversion	0	Yes	Moderate	Yes	Probably damaging	Pathologic
4363 T>C	N/A	Anticodon loop of tRNA glutamine	Transition	0.62	No	High	N/A	Unknown	Non-pathologic
4385 A>G	N/A	In the TyC domain of the tRNA glutamine	Transition	0.62	No	High	N/A	Unknown	Non-Pathologic
4648 T>C	F60S	Outside the TM domain of ND2 gene	Transition	0	Yes	High	Yes	Probably damaging	Pathologic
5182 C>T	T238M	Outside the TM domain of ND2 gene	Transition	0	Yes	Low	No	Benign	Non-Pathologic
5843 A>G	N/A	In the D-loop of tRNA tyrosine	Transition	1.2	No	High	N/A	Unknown	Non-pathologic
6546 C>T	L215F	Outside the TM domain of COI gene	Transition	0.62	No	High	No	Benign	Non-pathologic
7877 A>C	K98Q	Outside the TM domain of COII gene	Transversion	0	Yes	High	No	Probably damaging	Pathologic
9103 T>C	F193L	Outside the TM domain of ATPase 6 gene	Transition	1.2	No	Moderate	No	Benign	Non-pathologic
9438 G>A	G78S	Outside the TM domain of COIII gene	Transition	3.14	No	High	No	Benign	Non-pathologic
11337 A>G	N193S	TM domain of ND4 gene	Transition	1.9	No	Low	No	Benign	Non-pathologic
12841 A>G	I169V	Outside the TM domain of ND5 gene	Transition	3.1	No	Low	No	Benign	Non-pathologic
13813 G>A	V493I	TM domain of ND5 gene	Transition	1.9	No	High	No	Benign	Non-pathologic

In the “Base substitution type” column, Transversion=A mutation in which a purine/pyrimidine replaces a pyrimidine/purine base pair or vice versa (G:C>T:A or C:G, or A:T>T:A or C:G); Transition=A mutation in which a purine/pyrimidine base pair is replaced with a base pair in the same purine/pyrimidine relationship (A:T>G:C or C:G>T:A). Controls (%)=percent of controls with this nucleotide substitution. Previous reports of sequence variants were found in the Human Mitochondrial Genome Database (MITOMAP database), GenBank, and Medline listed publications. Interspecies conservation was assessed using the Polymorphism Phenotyping (PolyPhen) database, which determines interspecies conservation for an altered amino acid by performing alignment with all available amino acid sequences for other species, and the Mamit-tRNA website when necessary. Protean predicts and displays secondary structural characteristics. “Yes”=nucleotide change will alter protein secondary structure; “No”=change will not alter secondary structure. PolyPhen prediction of pathogenicity was assessed using the PolyPhen database. “Probably damaging” constitutes a high confidence of affecting protein function or structure. “Possibly damaging” reflects a likelihood of affecting protein function or structure, while “Benign” changes most likely lack phenotypic effect. “Unknown” means that PolyPhen could not make a prediction due to lack of data. None of these nucleotide changes was heteroplasmic. Summary; see Prediction of Pathogenicity in Methods. TM=transmembrane. N/A=not applicable because the database is not designed to predict this type of sequence change. Reported haplogroup specific NS sequence changes were excluded from Table 2 and further analysis.

Two of these mtDNA sequence alterations (nt 3833 and nt 4648) cause amino acid (AA) changes (L176Q in ND1 and F60S in ND2, respectively) in Complex I (NADH: ubiquinone oxidoreductase), which is a major component of the oxidative phosphorylation machinery responsible for producing much of the ATP required by cells. The third potentially pathological mtDNA nucleotide change (nt 7877) causes an AA change (K98Q) in the COII gene of Complex II (succinate: ubiquinone oxidoreductase), which performs a key step in the citric acid cycle in which succinate is dehydrogenated to fumarate and electrons are donated to ubiquinone in the mitochondrial inner membrane. Mutations in either Complex I or Complex II may impair the electron transfer process and result in decreased ATP production and/or accumulation of reactive oxygen species, although this cannot be proven without investigating mitochondrial cybrids. The nt 3833 nucleotide change replaces the AA Leucine (hydrophobic) with Glutamine (neutral), changing the hydrophilicity index and possibly altering protein structure flexibility. The nt 4648 nucleotide change replaces Phenylalanine (hydrophobic) with Serine (neutral), changing the hydrophilicity index and possibly causing a loss of Alpha region with introduction of an aberrant Beta region at that AA location. The nt 7877 nucleotide change replaces Lysine (hydrophilic) with Glutamine (neutral), altering the hydrophilicity index and changing protein flexibility.

Table 3 details mtDNA sequence changes in each patient. Seventeen patients (Patients 1–17) had no NS mtDNA sequence changes listed in Table 2, while nine (Patients 18–26) had NS sequence changes predicted to be non-pathologic. The three potentially pathologic mtDNA sequence changes were present in three different patients (Patients 27–29).

**Table 3:** mtDNA and mitochondrial respiratory changes in PEG patients.

**Patient**	**Nucleotide Change(s)**	**Relative mtDNA Content**	**MRA**
1	None	1.2	21.6
2	None	1.35	21.8
3	None	1.2	21.9
4	None	1.9	19.6
5	None	1.4	20.4
6	None	1.3	21.6
7	None	1.2	21.6
8	None	1.1	22.5
9	None	1.2	20.5
10	None	1.1	19.8
11	None	1.3	18.9
12	None	1.5	21.9
13	None	1.3	21.4
14	None	0.92	21.9
15	None	0.81	22.5
16	None	1.1	21.4
17	None	1.08	19.8
18	5182	0.8	21.4
19	4363	1.2	20.4
20	4385, 12841	1.2	21.5
21	9438	1.6	20.8
22	5843	1.2	20.2
23	9103, 13813	0.83	21.9
24	9103	0.82	21.8
25	11337	1.2	20.4
26	4385, 6546	1.09	18.6
27	3833	0.85	19.8
28	7877	1.2	20.8
29	4648	1.4	21.4

PEG patients organized according to characteristics of “Nucleotide Change(s)” from Table 2 for each patient. Patients 1–17 had no noteworthy mtDNA changes (see Methods), while patients 18–26 had NS mtDNA changes not thought to be pathologic, and patients 27–29 had NS mtDNA changes thought likely to be pathologic (see Methods). Relative mtDNA Content=ratio of ND1 to β-actin (see Methods). MRA=Mitochondrial Respiratory Activity (see Methods).

### Relative mitochondrial DNA content and mitochondrial functional testing

Table 3 also details relative mtDNA content and MRA by patient. Mean relative mtDNA content in PEG patients (1.18 ±0.25; 95% CI 1.09–1.28) was not significantly different from controls (1.18 ±0.17; 95% CI 1.12–1.25; p=0.98). Likewise, MRA in PEG patients (20.97 ±1.03; 95% CI 20.58–21.36) was not significantly different from controls (21.25 ±0.80; 95% CI 21.03–21.25; p=0.177).

## Discussion

We evaluated 29 patients who had pseudoexfoliation material in the anterior chamber and met anterior segment, fundoscopic and visual field criteria that define PEG. None of the patients met criteria for POAG or PACG, and none had the clinical characteristics of other spontaneous optic neuropathies associated with mitochondrial abnormalities such as Leber hereditary optic neuropathy [31]. As a group, they had relatively advanced ophthalmologic disease with 15 being legally blind in one eye, 2/3 having severe central or peripheral visual field loss, and almost half requiring a glaucoma surgical procedure. Compared to a group of POAG patients evaluated previously in a similar fashion [9], these PEG patients had slightly worse VA and slightly more surgical interventions.

No patient had a novel or previously described mutation in one of the nuclear genes investigated here. *MYOC, OPTN, WDR36,* and *CYP1B1* have all been identified in a relatively small percentage of patients with various types of glaucoma, and it remains possible that these genes are abnormal with a small percentage of PEG patients. This is the first time that these genes, or *OPA1* and *OPA3,* have been completely sequenced in patients with PEG, but a report of a recent genome-wide screen of Icelandic and Swedish populations [32] also failed to identify an association between PEG and the nuclear genes evaluated here. The study did detect an association of PEG with two nonsynonymous SNPs in exon 1 of the *LOXL1* gene. PES has a familial association [7,8] and may have a nuclear genetic predisposition due to mutation of the *LOXL1* gene or to other nuclear genes not yet identified.

Mitochondrial function is multifaceted, and various mitochondrial abnormalities have variable, and currently unpredictable, effects on different tissues. This study evaluated three mitochondrial parameters (the entire mitochondrial DNA coding region sequence, relative mtDNA content, and mitochondrial respiratory activity) that address mitochondrial structure and function from three largely unrelated perspectives. This evaluation is not comprehensive, but these mitochondrial parameters taken together have the potential of detecting disparate mitochondrial abnormalities. Abnormalities in these three mitochondrial parameters have proven to be aligned in certain (e.g., LHON-like optic neuropathies [25]), but not all (e.g., POAG [9]), spontaneous optic neuropathies. In the PEG patients reported here, only three patients (10.3%) had mtDNA sequence changes that were predicted to be pathologic, a smaller representation of mtDNA abnormalities than found in several other spontaneous optic neuropathies [9,25,33,34]. Neither relative mtDNA content nor MRA was changed in PEG patients compared to matching controls. These results imply that the nuclear and mitochondrial factors investigated here are likely less important than anatomic and dynamic disturbances consequent to deposition of pseudoexfoliation material in the anterior chamber.

These largely negative results in PEG patients are similar to data documented in patients with PACG [35]. Although PEG and PACG have different mechanisms, the intermediate result is elevation of IOP which, in turn, is a likely primary cause of optic nerve injury. This mechanism contrasts with the molecular and biochemical abnormalities suspected in POAG [36], just as the PEG results reported here contrast dramatically with the substantial mitochondrial abnormalities observed in a group of POAG patients [9], highlighting the potential importance of mitochondrial malfunction as a metabolic risk factor for POAG. We report a relatively small number of Middle Eastern Arabian patients, and these genetic and mitochondrial studies should be confirmed in other ethnicities and extended to other types of glaucoma.
